# Prevalence of masked obesity and its association with body composition and lifestyle factors in young adults: a cross-sectional analysis

**DOI:** 10.3389/fpubh.2026.1854278

**Published:** 2026-10-21

**Authors:** Yimei Li, Tao Zhang, Luowen Chen

**Affiliations:** Department of Health Management, Fujian Health College, Fuzhou, China

**Keywords:** body composition, lifestyle factors, masked obesity, muscle-to-fat ratio, young adults

## Abstract

**Objectives:**

This study aims to investigate the prevalence of masked obesity among vocational college students, evaluate the predictive efficacy of the muscle-to-fat ratio (MFR), and explore the associations between masked obesity and lifestyle factors.

**Methods:**

A cross-sectional study was conducted with 320 students from Fujian Health College. A self-developed structured questionnaire was used to collect data on demographic characteristics and health-related behaviors. Body composition indicators were measured via a bioelectrical impedance analysis (InBody 770c). According to different combinations of body mass index (BMI) and body fat percentage (BF%), they were divided into the following 8 groups: (1) under weight with low body fat group (UW-L); (2) Under weight with high body fat group (UW-H); (3) Normal weight with low body fat group (NW-L); (4) Normal weight with high body fat group (NW-H); (5) Overweight with low body fat group (OW-L); (6) Overweight with high body fat group (OW-H); (7) Obese with low body fat group (OB-L); (8) Obese with high body fat group (OB-H). Among them, the two types of UW-H and NW-H together constitute masked obesity.

**Results:**

The prevalence of masked obesity was as high as 33.9% in the normal-weight population, and 5.8% in the underweight population. MFR demonstrates better diagnostic efficacy in the detection of masked obesity (AUC = 0.861), and is superior to BMI. After adjusting for confounding factors, excessive sleep duration was associated with masked obesity (OR = 6.887, 95% CI: 1.417–33.473), while no significant association was found between sleep quality and physical activity and masked obesity.

**Conclusions:**

Compared with the traditional obesity index BMI, MFR is more effective in identifying masked obesity in young adults.

## 1 Introduction

Obesity is a major risk factor for the occurrence and progression of various chronic diseases and has become one of the world's public health problems threatening human health (1). Research has established a close link between obesity and the development of metabolic syndrome, cardiovascular disease, sleep apnea syndrome, and cancer. Notably, obesity in young individuals not only opens an “early channel” to chronic disease but also poses more long-term hazards (2–4). The Body Mass Index (BMI) is widely used as a screening tool for obesity due to its simplicity. However, because BMI cannot accurately distinguish between fat and muscle mass, it often leads to missed diagnoses among individuals with masked obesity. masked obesity refers to a condition where individuals have a normal or low BMI but an excessive body fat percentage (BF%), accompanied by a deficiency in lean body mass (5).

Most of the existing studies focus on traditional fat indicators such as BMI or BF%, while seriously neglecting the dynamic interaction between muscle and fat. The muscle-to-fat ratio (MFR), as a comprehensive indicator reflecting the balance between lean body mass and adipose tissue, has been proven to be a superior predictor of metabolic health status (6–8). However, there is still a lack of evidence regarding the value of MFR in the screening of masked obesity, especially for college students who are in a period of rapid lifestyle change.

Although MFR has significant clinical significance, its research on masked obesity is still relatively insufficient, especially among young people. Therefore, this study aims to investigate the prevalence of masked obesity among vocational college students, evaluate the predictive efficacy of the MFR, and explore the associations between masked obesity and lifestyle factors. The aim is to provide a basis for targeted intervention measures for the early detection and prevention of obesity in young adults.

## 2 Methods

### 2.1 Study population

This study adopted a cross-sectional research design and enrolled 320 students (100 males and 220 females) from Fujian Health College. Exclusion criteria were as follows: (1) Long-term use of corticosteroids, endocrine drugs or immunosuppressive drugs; (2) Presence of metabolism-related diseases, including malignant tumors, kidney dysfunction or hyperthyroidism; (3) Missing key variables such as BF % and BMI. The study was approved by the ethics committee of Fujian Health College, and informed consent was obtained from all participants.

### 2.2 Measures

#### 2.2.1 Questionnaire

Structured questionnaire was used to conduct the investigation, with a total of 43 closed-end questions, which mainly included information on demographic characteristics (gender, age, etc.) and health-related behaviors (smoking, drinking, physical activity, and sleep).

#### 2.2.2 Physical measurement

Body weight and height were measured with the patient barefoot and wearing light clothes. Body composition indicators were measured via a bioelectrical impedance analysis (BIA) (InBody 770c). Obesity-related data included BF%, Waist-to-hip ratio (WHR) and visceral fat area (VFA), and muscle-related data included soft lean mass (SLM) and appendicular skeletal muscle masss (ASLM). BMI (kg/m^2^) was defined as weight in kilograms divided by square of the height in meters. Skeletal muscle index (SMI, kg/m^2^) was defined as the appendicular skeletal muscle mass in kilograms divided by square of the height in meters (9). MFR was calculated by whole-body soft lean mass divided by fat mass (10).

#### 2.2.3 Diagnostic criteria

Underweight was defined as BMI < 18.5 kg/m^2^; normal weight as 18.5– < 24.0 kg/m^2^; overweight as 24.0– < 28.0 kg/m^2^; and obesity as ≥28.0 kg/m^2^. Participants were categorized into a low body fat group (men: BF < 25%; women: BF < 30%) and a high body fat group (men: BF ≥ 25%; women: BF ≥ 30%) (11). According to the classification of extended normal weight obesity (eNWO) (12), the participants were divided into the following 8 groups: (1) under weight with low body fat group (UW-L); (2) Under weight with high body fat group (UW-H); (3) Normal weight with low body fat group (NW-L); (4) Normal weight with high body fat group (NW-H); (5) Overweight with low body fat group (OW-L); (6) Overweight with high body fat group (OW-H); (7) Obese with low body fat group (OB-L); (8) Obese with high body fat group (OB-H). Normal-weight obesity refers to individuals with a BMI within the normal range but a BF% exceeding sex-specific thresholds, corresponding to the NW-H group. Underweight with high body fat refers to individuals with a BMI below the normal range and a BF% exceeding sex-specific thresholds, corresponding to the UW-H group. Besides the overweight and obese populations, UW-H and NW-H also demonstrate excessive fat accumulation. Therefore, Masked obesity is operationally defined as individuals with a BMI below the overweight threshold but a significantly elevated BF%, corresponding to the UW-H and NW-H groups (13, 14). Physical activity levels were assessed using the short form of the International Physical Activity Questionnaire (IPAQ). Participants were stratified into low, moderate, and high activity groups based on tertiles. Sleep duration < 6 h per day was defined as insufficient sleep, 6–8 h as appropriate sleep, and > 8 h as excessive sleep (15). We used the Pittsburgh Sleep Quality Index (PSQI) to evaluate the sleep quality of the participants, and the cutoff value for defining poor sleep quality is >5 points (16).

### 2.3 Statistical analysis

The SPSS 25.0 software (IBM, Armonk, NY, USA) was used to perform statistical analysis. Continuous variables were expressed as mean ± standard deviation or median (P25–P75), and categorical variables were presented as count and percentage. For subgroups with *n* < 5, only descriptive statistics (individual values or median with range) are reported. Differences in the baseline characteristics were analyzed by ANOVA test, Kruskal–Wallis test and the chi squared test. Multiple logistic regression analysis was employed to further investigate the factors influencing masked obesity. Categorical variables were compared using Fisher's exact test when expected cell counts were < 5. The area under the curve (AUC) of MFR, BMI and SMI at the diagnosis of masked obesity were calculated using the receiver operating characteristic (ROC) analyses. *P* < 0.05 was considered statistically significant.

## 3 Results

### 3.1 Study population characteristics

Compared with females, males were significantly higher in terms of smoking, drinking, height, weight, BMI, SLM, ASLM, SMI and MFR (all *P* < 0.05). While females were significantly higher in terms of WHR, BF% and VFA. The results were statistically significant (all *P* < 0.05) as shown in Table 1.

**Table 1:** Basic characteristics of participants.

Variable	Men	Female	*P*
Age (years old)	19.00 (18.00–20.00)	19.00 (18.00–19.00)	0.019^*^
Smoker	21 (20.8)	5 (2.3)	< 0.001^*^
Alcohol	26 (25.7)	24 (11.0)	0.001^*^
Height (cm)	174.00 (170.00–178.50)	161.00 (158.00–165.00)	< 0.001^*^
Weight (kg)	64.00 (57.50–73.45)	53.60 (47.90–60.10)	< 0.001^*^
WHR	0.81 (0.78–0.85)	0.82 (0.80–0.87)	0.029^*^
BMI	21.50 (19.45–24.15)	20.70 (18.50–23.00)	0.037^*^
BF%	18.53 ± 7.61	29.56 ± 6.59	< 0.001^*^
VFA (cm^2^)	42.30 (27.15–63.80)	63.70 (48.60–92.50)	< 0.001^*^
SML	49.80 (45.50–55.25)	35.20 (32.80–38.50)	< 0.001^*^
Appendicular skeletal muscle mass	22.61 (20.20–25.54)	15.02 (13.59–16.84)	< 0.001^*^
SMI	7.50 (7.00–8.00)	5.80 (5.40–6.20)	< 0.001^*^
MFR	4.62 (3.29–6.14)	2.30 (1.84–2.87)	< 0.001^*^

WHR, waist-to-hip ratio; BMI, body mass index; BF%, body fat percentage; VFA, visceral fat area; SLM, soft lean mass; SMI, skeletal muscle index; MFR, muscle-to-fat ratio.

Data are expressed as mean ± standard deviation for continuous variables, and as percentages (%) for categorical variables. Differences in the baseline characteristics were analyzed by ANOVA test, Kruskal–Wallis test and the chi squared test.

^*^P < 0.05.

### 3.2 Prevalence of masked obesity and body composition characteristics across groups

As shown in Table 2, there was a significant difference in the prevalence of masked obesity among participants with different BMI phenotypes (*P* < 0.001). Notably, masked obesity was absent in the overweight and obese populations. The prevalence of masked obesity was 5.8% in the underweight group, while it was substantially higher in the normal weight group, reaching 33.9%. The number of participants classified and stratified according to the expanded normal weight obesity category is shown in Supplementary Table 1.

**Table 2:** Comparison of the prevalence of masked obesity among participants with different BMI phenotypes.

BMI phenotype	Prevalence of masked obesity *N* (%)	*P*
Underweight	4 (5.8%)	< 0.001^*^
Normal weight	64 (33.9)	
Overweight	—	
Obesity	—	

Prevalence of masked obesity: 33.9% in normal–weight group, 5.8% in underweight group.

^*^P < 0.05.

Table 3 presents the comparison of body composition among different study groups. Significant differences were observed across the groups in fat indicators, including BMI, BF%, WHR, and VFA, as well as muscle indicators SLM and SMI. Overall, with comparable or slightly higher BMI, the high body fat groups (UW-H, NW-H, OW-H, and OB-H) exhibited significantly higher WHR and VFA compared to the low body fat groups (UW-L, NW-L, OW-L, and OB-L). Notably, individuals with masked obesity (UW-H and NW-H) demonstrated markedly elevated BF% (31.33 ± 1.04, 33.52 ± 2.56, *P* < 0.001) and VFA [66.40 (51.23–67.48), 87.70 (76.93–99.05), *P* < 0.001] (Table 3a), surpassing even some overweight and obese individuals in fat indicators despite having non-excessive body weight. Consistent with these findings, the muscle mass indicators (Table 3b) showed a negative correlation with body fat levels. The low body fat group maintained higher SLM and SMI values. It is worth noting that the individuals with masked obesity had significantly lower SLM [29.85 (27.13–32.88), 35.20 (33.13–38.00), *P* < 0.001] and SMI [4.90 (4.68–5.20), 5.85 (5.60–6.08), *P* < 0.001].

**Table 3a:** Comparison of fat indicators among different groups of study populations.

Group	BMI/(kg·m^2^)	BF%	WHR	VFA
UW-L	17.40 (16.70–17.90)	19.31 ± 5.70	0.79 (0.77–0.81)	36.50 (28.70–42.95)
UW-H	18.30 (17.98–18.40)	31.20 (30.43–32.35)	0.81 (0.76–0.84)	66.40 (51.23–67.48)
NW-L	20.50 (19.30–21.60)	21.90 ± 5.88	0.81 (0.79–0.83)	51.20 (40.60–59.20)
NW-H	22.00 (21.03–23.15)	33.52 ± 2.56	0.85 (0.82–0.87)	87.70 (76.93–99.05)
OW-L	24.40 (24.18–24.93)	18.77 ± 3.87	0.85 (0.80–0.88)	61.20 (50.18–69.38)
OW-H	25.50 (24.85–26.45)	34.73 ± 5.13	0.89 (0.87–0.91)	106.90 (92.35–127.93)
OB-L	28.30	24.30	0.91	103.00
OB-H	31.60 (29.55–35.20)^**^	39.42 ± 5.67^**^	0.94 (0.92–1.00)^**^	167.00 (142.15–191.40)^**^

BMI, body mass index; BF%, body fat percentage; WHR, waist-to-hip ratio; VFA, visceral fat area.

Differences in the baseline characteristics were analyzed by ANOVA test, Kruskal–Wallis test and the chi squared test.

^**^P < 0.001.

**Table 3b:** Comparison of muscle indicators among different groups of study populations.

Group	SLM	SMI
UW-L	33.10 (30.80–37.45)	5.40 (5.10–6.05)
UW-H	29.85 (27.13–32.88)	4.90 (4.68–5.20)
NW-L	40.10 (35.55–49.00)	6.50 (5.80–7.40)
NW-H	35.20 (33.13–38.00)	5.85 (5.60–6.08)
OW-L	59.00 (58.30–64.65)	8.40 (8.10–8.93)
OW-H	41.90 (39.83–45.75)	6.85 (6.50–7.25)
OB-L	67.40	9.00
OB-H	51.10 (44.25–59.20)^**^	8.20 (7.40–8.80)^**^

SLM, soft lean mass; SMI, skeletal muscle index.

^**^P < 0.001.

Table 3c presents the comparison of MFR among different study groups. The results indicated that within the same BMI categories, the high body fat groups (UW-H, NW-H, OW-H, and OB-H) exhibited significantly lower MFR values compared to the low body fat groups (UW-L, NW-L, OW-L, and OB-L), with statistical significance (*P* < 0.001). Of particular note is that individuals with masked obesity (UW-H and NW-H) demonstrated markedly lower MFR [2.07 (1.95–2.14), 1.90 (1.72–2.04), *P* < 0.001], which was not only lower than that of the low body fat groups within the same BMI categories but also lower than that of the low body fat groups in the overweight and obese categories.

**Table 3c:** Comparison of MFR among different groups of study populations.

Group	MFR
UW-L	3.67 (3.09–4.95)
UW-H	2.07 (1.95–2.14)
NW-L	3.13 (2.54–4.62)
NW-H	1.90 (1.72–2.04)
OW-L	3.87 (3.34–5.03)
OW-H	1.82 (1.51–2.12)
OB-L	2.91
OB-H	1.42 (1.23–1.69)^**^

MFR, muscle-to-fat ratio.

^**^P < 0.001.

### 3.3 Predictive value of MFR and BMI for masked obesity

Receiver operating characteristic (ROC) curve analysis was performed to evaluate the predictive performance of MFR and BMI for masked obesity. As shown in Figure 1, MFR demonstrated excellent discriminatory ability, with an area under the curve (AUC) of 0.861 (95% CI: 0.821–0.902; *P* < 0.001), as assessed by the Youden index, was 2.22 (Figure 1A). In contrast, BMI exhibited a relatively lower predictive value, with an AUC of 0.608 (95% CI: 0.548–0.668; *P* = 0.006). Based on the Youden index, the optimal cutoff value for diagnosing masked obesity is 20.65 (Figure 1B). Furthermore, the AUC of SMI was 0.696 (95% CI: 0.641–0.752; *P* < 0.001). According to the Youden index, the optimal cutoff value for diagnosing masked obesity was 6.25 (Figure 1C).

**Figure 1:**
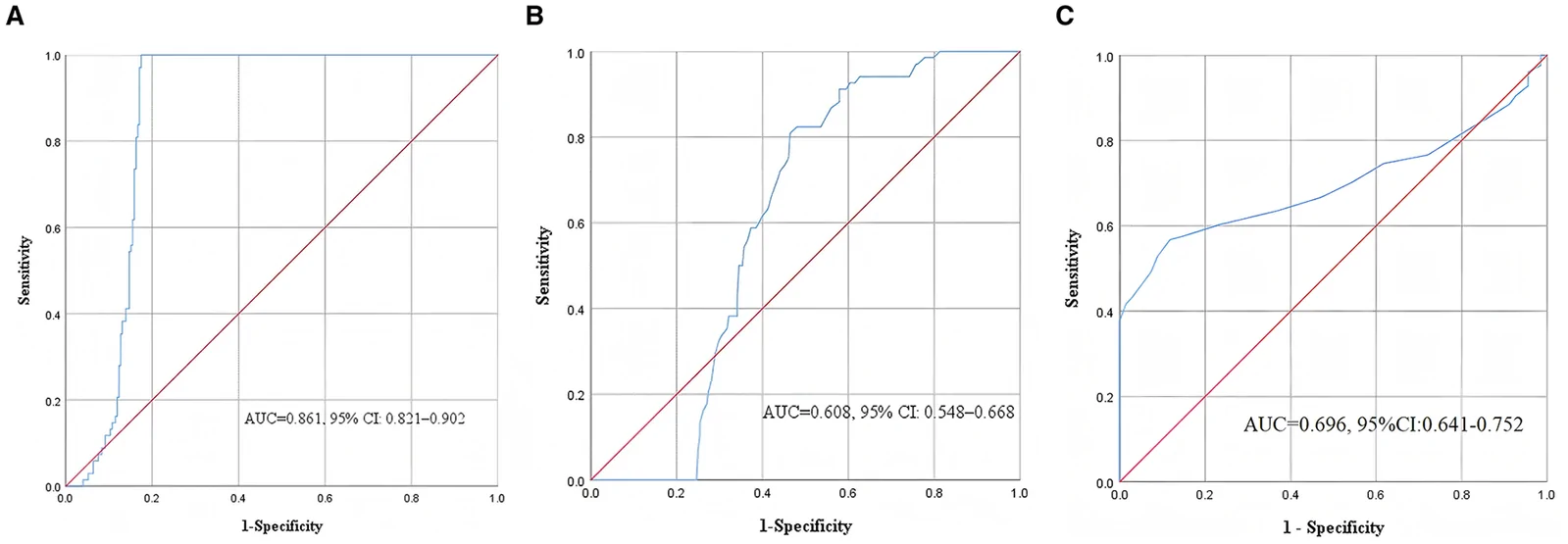
The area under the ROC curve of muscle-to-fat ratio (MFR), body mass index (BMI), and skeletal muscle index (SMI) detection for the screening of masked obesity: **(A)** is the ROC curve for the screening of masked obesity by MFR, **(B)** is the ROC curve for the screening of masked obesity by BMI, **(C)** is the ROC curve for the screening of masked obesity by SMI. MFR demonstrated an area under the curve (AUC) of 0.861 (95% CI: 0.821–0.902), with an optimal cutoff of 2.22. BMI showed a lower AUC of 0.608 (95% CI: 0.548–0.668). SMI demonstrated an AUC of 0.696 (95% CI: 0.641–0.752).

### 3.4 Logistic regression of influencing factors of masked obesity

Table 4 presents the results of the multivariate logistic regression analysis to further investigate the factors influencing masked obesity. Compared with appropriate sleep, excessive sleep was associated with masked obesity (OR = 6.887, *P* = 0.017) after adjusting for potential confounding factors (including sex, age, smoking, drinking, and height). Furthermore, no significant associations were observed between physical activity, poor sleep quality and masked obesity (*P* > 0.05).

**Table 4:** Analysis of factors affecting masked obesity.

Variable	Multivariate logistic regression analysis	
	OR (95% Cl)	*P*
Sleep duration		
Insufficient sleep	1.186 (0.286–4.920)	0.814
Appropriate sleep	—	0.057
Excessive sleep	6.887 (1.417–33.473)	0.017^*^
Sleep quality		
Poor sleep quality	1.500 (0.572–3.931)	0.410
Physical activity		
Low	—	0.702
Moderate	1.257 (0.539–2.931)	0.596
High	0.858 (0.344–2.136)	0.741

Insufficient sleep, < 6 h per day; appropriate sleep, 6–8 h per day; excessive sleep, >8 h per day.

Multiple logistic regression analysis was employed to determine whether lifestyle behaviors were independently associated with masked obesity. Adjusted for sex, age, height, smoking, and drinking.

^*^P < 0.05.

## 4 Discussion

Based on a cross-sectional survey, this study explored the epidemiological characteristics of masked obesity in higher vocational colleges, as well as its association with body composition indicators and lifestyle factors. The research findings are as follows: firstly, the prevalence of masked obesity was as high as 33.9% in the normal-weight population, and 5.8% in the underweight population. Secondly, MFR demonstrates better diagnostic efficacy in the detection of masked obesity (AUC = 0.861), and is superior to BMI. Thirdly, after adjusting for confounding factors, excessive sleep was found to be significantly positively correlated with masked obesity (OR = 6.887, 95% CI: 1.417–33.473).

Firstly, our study reveals that the prevalence of high BF% reaches 33.9% among individuals with normal BMI, with an additional 5.8% observed in the underweight population, suggesting that a large number of individuals with potential metabolic risks are being undiagnosed. This result also highlights the limitations of using BMI for obesity screening (17). It is worth noting that even underweight young adults may have high body fat levels, suggesting that the “skinny fat” phenomenon has emerged as a significant public health concern. Our data show that individuals with masked obesity exhibit a distinct body composition phenotype, which can be described as “sarcopenic obesity”, characterized by significantly reduced SMI and SLM, along with abnormally elevated VFA (18, 19). These data suggest that fat accumulation is predominantly located in the abdominal and visceral regions, that is, “central obesity” (20, 21). Visceral fat contributes to insulin resistance via chronic low-grade inflammation and ectopic lipid deposition, thereby increasing the risk of type 2 diabetes, dyslipidemia, and hypertension (22). Of particular interest is that the VFA of masked obesity individuals even exceeds that of some overweight/obese individuals, which vividly illustrates the metabolic paradox of being ‘thin outside but fat inside.' This body composition indicates that these young people with a normal or even low BMI but high BF% may already be in the early stage of developing metabolic syndrome.

Secondly, our research results indicate that MFR is a stronger predictor of masked obesity than BMI. The ROC curve analysis revealed that the AUC of MFR is as high as 0.861, significantly superior to BMI. Based on the Youden index, we determined that the optimal cut-off value of MFR is 2.22. This suggests that BMI cannot accurately distinguish between body fat and lean body mass. In addition, we evaluated the predictive performance of the muscle indicator SMI without fat components in the case of masked obesity. The SMI alone yielded an AUC of 0.701 (95% CI: 0.645–0.756), which was statistically significant. The independent predictive value of the SMI supports the importance of muscle composition in masked obesity. MFR, which can reflect the “dynamic balance between muscle and fat,” can more comprehensively reflect metabolic health status and assess health risks. Consistent with previous studies, the MFR is regarded as a “Load-Capacity Index”, capturing the physiological interplay between metabolic capacity (skeletal muscle mass) and metabolic load (adipose tissue) (10, 23). Aligning with these findings, our study confirms that the MFR transcends the limitations of BMI, offering a more precise evaluation of masked obesity.

Finally, in the study on masked obesity and lifestyle, we found that after adjusting for confounders, excessive sleep was associated with masked obesity compared to the appropriate sleep. This U-shaped or J-shaped correlation between sleep and obesity has also been reported in other studies (24–26). Excessive sleep duration is to some extent a potential reflection of phenomena such as low energy expenditure and disrupted circadian rhythms (27). However, it is worth noting that the 95% CI (1.417–33.473) is wide, which is likely related to the smaller number of participants in the excessive sleep group. Further confirmation is needed through a study with a larger sample size in the future. Furthermore, as no significant association was observed between sleep quality and masked obesity in our regression models, we believe that while excessive sleep duration may promote the development of masked obesity, poor sleep quality does not act as a synergistic factor. It is worth noting that this study did not find a significant correlation between physical activity and masked obesity. This may be attributed to the “sedentary learning mode” commonly seen among college students, in which sleep serves as a more prominent physiological regulatory factor, while physical activity fails to exert its intended metabolic protective effect.

The strengths of this study lies in its examination of the prevalence of masked obesity among the young population, the use of objective measurements of body composition to avoid potential biases associated with self-reported anthropometric indicators, and the comprehensive assessment of lifestyle factors. Importantly, our research results have expanded the latest understanding of the clinical value of MFR. However, several limitations should be acknowledged. First, due to the cross-sectional design, causal relationships cannot be established. Second, the reliance on self-reported questionnaires for sleep duration and physical activity may introduce recall bias. Thirdly, the relatively small sample size may have obscured other potential interactions. Finally, the ROC comparison between MFR and BMI may be partially influenced by mathematical coupling. However, the MFR also reflects lean mass, a key component of masked obesity. Further research is needed to confirm its clinical application value.

In conclusion, this study highlights the limitations of the traditional obesity indicator BMI as the sole indicator for assessing the obesity risk of young people. MFR has been proven to be a highly valuable tool for screening for masked obesity. More importantly, the study suggests that the focus of public health intervention should not be limited to increasing physical activity alone, more attention should be paid to sleep and avoiding prolonged sitting, which holds significant practical implications for preventing the “thin on the outside but fat on the inside” phenomenon among college students.

## Data Availability

The raw data supporting the conclusions of this article will be made available by the authors, without undue reservation.
