# Supplementary material for: Prevalence of masked obesity and its association with body composition and lifestyle factors in young adults: a cross-sectional analysis
Source: Front Public Health. 2026 Oct 21;14:1854278. doi: 10.3389/fpubh.2026.1854278 (PMC13731359; doi:10.3389/fpubh.2026.1854278)
Supplement: Supplementary file 1 [file Table_1.docx]

Supplemental table 1 Number of participants stratified by the extended normal-weight obesity classification

|  | N |
| --- | --- |
| UW-L | 65 |
| UW-H | 4 |
| NW-L | 125 |
| NW-H | 64 |
| OW-L | 10 |
| OW-H | 30 |
| OB-L | 1 |
| OB-H | 21 |
